# PlutoNet: An efficient polyp segmentation network with modified partial decoder and decoder consistency training

**DOI:** 10.1049/htl2.12105

**Published:** 2024-12-13

**Authors:** Tugberk Erol, Duygu Sarikaya

**Affiliations:** ^1^ Computer Engineering Graduate School of Natural and Applied Sciences Gazi University Ankara Türkiye; ^2^ School of Computer Science University of Leeds Leeds United Kingdom

**Keywords:** computer vision, convolutional neural nets, image segmentation, learning (artificial intelligence), medical image processing, neural nets

## Abstract

Deep learning models are used to minimize the number of polyps that goes unnoticed by the experts and to accurately segment the detected polyps during interventions. Although state‐of‐the‐art models are proposed, it remains a challenge to define representations that are able to generalize well and that mediate between capturing low‐level features and higher‐level semantic details without being redundant. Another challenge with these models is that they are computation and memory intensive, which can pose a problem with real‐time applications. To address these problems, PlutoNet is proposed for polyp segmentation which requires only 9 FLOPs and 2,626,537 parameters, less than 10% of the parameters required by its counterparts. With PlutoNet, a novel *decoder consistency training* approach is proposed that consists of a shared encoder, the *modified partial decoder*, which is a combination of the partial decoder and full‐scale connections that capture salient features at different scales without redundancy, and the auxiliary decoder which focuses on higher‐level semantic features. The *modified partial decoder* and the auxiliary decoder are trained with a combined loss to enforce consistency, which helps strengthen learned representations. Ablation studies and experiments are performed which show that PlutoNet performs significantly better than the state‐of‐the‐art models, particularly on unseen datasets.

## Graphical abstract

We propose PlutoNet for polyp segmentation, which significantly reduces computational complexity with only 9 FLOPs and 2.6 million parameters, less than 10% of what other models require. It introduces a novel decoder consistency training approach using a shared encoder, a modified partial decoder to capture multi‐scale features, and an auxiliary decoder focused on high‐level semantics. A combined loss function then enforces consistency between the more salient and the higher level, semantic features, enhancing the model's learned representations.

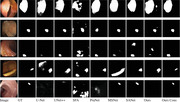

## 1 INTRODUCTION

According to the World Health Organisation (WHO), colon cancer is the third most common and the second most deadly cancer, accounting for approximately 10% of all cancer cases [1]. Polyps in the colon can turn into cancerous cells if not removed with early intervention. Studies show that during colonoscopy, depending on their type and size, 14–30% of polyps go unnoticed by the experts [2]. Deep learning models are used to minimize the number of polyps that go unnoticed by the experts and to accurately segment the detected polyps during these interventions. Although state‐of‐the‐art models perform well, they are computation and memory intensive; they require high computation and too many parameters, which can pose a problem with real‐time applications. It also remains a challenge for these models to generalize to unseen datasets and different domains.

In this work, we propose a novel segmentation model titled PlutoNet, which requires only 9 FLOPs and 2,626,537 parameters while outperforming state‐of‐the‐art models on several datasets. We propose a novel *consistency training approach*, which ensures a balance between the low‐level salient details at different scales learned through the *modified partial decoder* and the more relevant higher‐level semantic features learned through the auxiliary decoder. Enforcing consistency through a combined loss helps strengthen learned representations, and improves our model's generalizability on unseen datasets and different domains.

**FIGURE 1:**
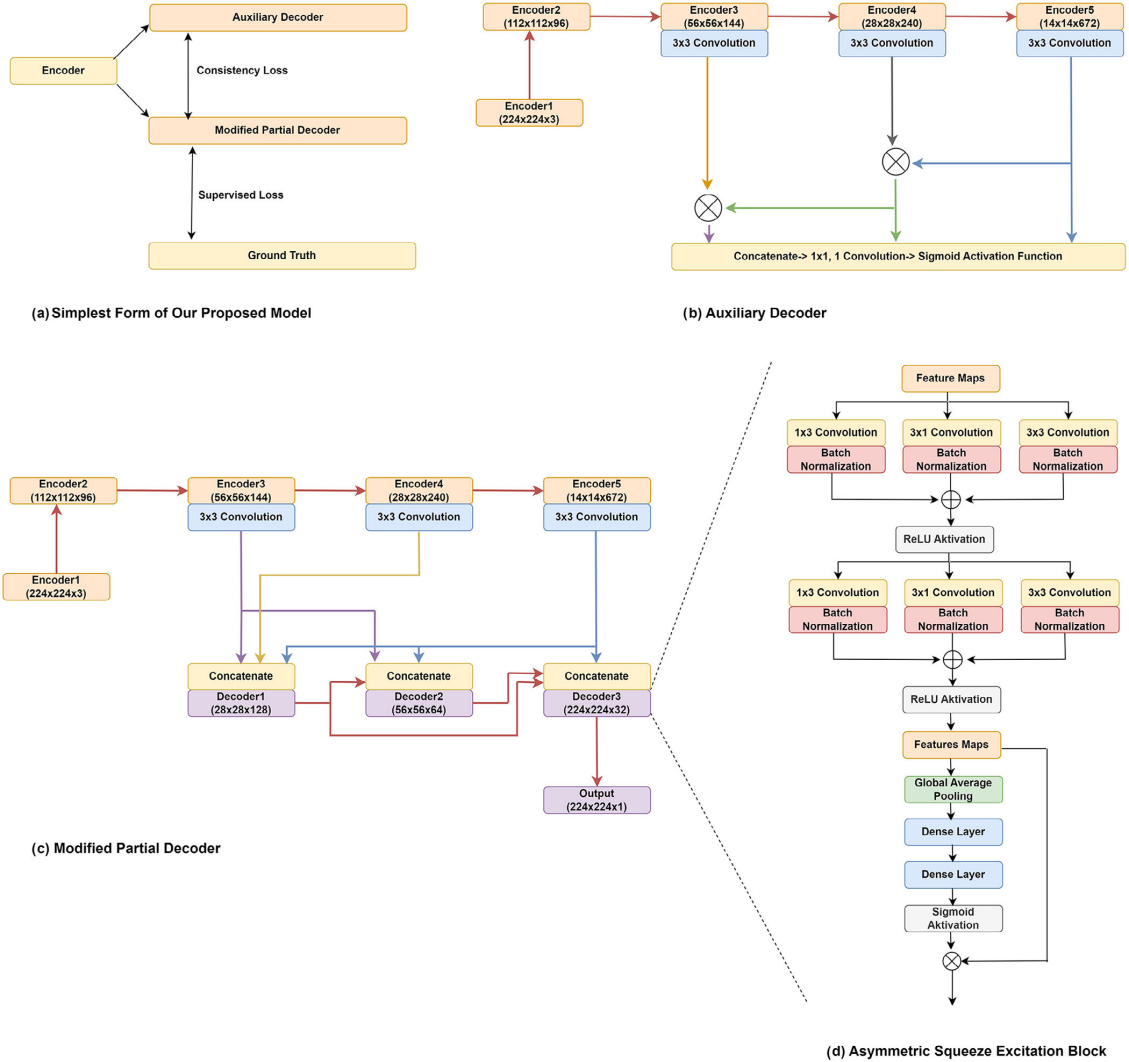
The overview of PlutoNet. In the top left corner (a), a high‐level representation of our proposed model is shown. In PlutoNet, a shared encoder, the *modified partial decoder*, and the auxiliary decoder are trained with a combined loss to enforce consistency. In the top right corner (b), the auxiliary decoder is shown. It carries out element‐wise multiplication of higher‐level encoders and then concatenates them. In the bottom left corner (c), the *modified partial decoder*, which is a combination of the partial decoder and full‐scale connections is shown. In the bottom right corner (d), the details of the decoder layers which use a combination of asymmetric convolutions and a squeeze and excitation block are shown.

PlutoNet architecture adopts a lightweight encoder‐decoder structure [3]. As repeated feature down‐sampling may cause small polyps to be easily degraded [4], state‐of‐the‐art models carry as much low and high‐level information as possible through skip connections. However, higher‐level encoder layers are shown to carry both low‐level and high‐level features [5], so using skip connections densely leads to redundant information and increases the number of parameters required. To address this, we use *modified partial decoder*, which is a combination of partial decoder [5] and full‐scale connections [6], and extend on the work proposed by Erol et al. [7]. Using *modified partial decoder*, we are able to reduce the number of parameters by ignoring skip connections to the low‐level features which may be redundant. Polyps in colonoscopy images have varying sizes, appearances, and aspect ratios. To handle these variations, we use asymmetric convolutions. We increase the representation of the more relevant features by weighting each feature map using a squeeze and excitation block following the findings of [7]. Our ablation studies with *modified partial decoder* shows that although each added component (asymmetric conv, squeeze excitation) increases the performance in segmentation metrics such as Dice and IoU, it decreases precision. This decrease in precision introduces uncertainty in the classification of certain regions as polyps or non‐polyps. In order to overcome this challenge, we propose a novel *consistency training approach*. With our *consistency training approach*, we enforce consistency by combining the loss of the *modified partial decoder*, which focuses more on learning salient features at different scales, and the auxiliary decoder, which focuses on the more relevant higher‐level semantic features. We show that this approach helps strengthen the learned representations. This way we are able to focus on the polyps and reduce false positive rates. An overview of our model is demonstrated in Figure 1.

We tested our model extensively for the segmentation of polyps in colonoscopy and endoscopy images on five different public datasets. We trained our model with Kvasir [2] and CVC‐ClinicDB [8] datasets. In addition to testing our model on these datasets, we tested it on the unseen ETIS [9], EndoScene [10], and CVC‐ColonDB [11] datasets. It should be noted that ETIS is a dataset consisting of images captured by capsule endoscopy and differs greatly in resolution. We outperformed the state‐of‐the‐art models with a Dice score of 82.9% on the ETIS dataset and 91.9% on the EndoScene dataset. Our experiments and ablation studies show that our model outperforms state‐of‐the‐art models and that it is able to generalize to several datasets and different domains. Moreover, PlutoNet requires less computation and only 2,626,537 parameters, which is far fewer than the state‐of‐the‐art models.

The main contributions of this paper are: (1) the novel *decoder consistency training* approach that ensures a balance between the salient details at different scales learned through the *modified partial decoder* and the more relevant higher‐level semantic features learned through the auxiliary decoder. Although conventionally used in unsupervised learning problems, we show that the representations learned through consistency training combining the loss of decoders focusing on more salient features and higher‐level semantic features perform well in segmentation tasks. To our knowledge, our study is the first to propose decoder‐level consistency training between two decoders with different goals on learning features. (2) We present PlutoNet which requires only 9 FLOPs and 2,626,537 parameters, which is far fewer than the state‐of‐the‐art models, about less than 10% of the parameters required by its counterparts. In order to achieve this, we adopt a lightweight encoder‐decoder structure [3] and extend on the *modified partial decoder* [7] that reduces the number of parameters by ignoring skip connections to the low‐level features which may be redundant. The auxiliary decoder adds only 200 parameters to our network architecture and is only needed for training. (3) We tested our model extensively for the segmentation of polyps in colonoscopy and wireless endoscopy images on five different public datasets. PlutoNet performs significantly better than the state‐of‐the‐art models, particularly on unseen datasets and datasets across different domains, which demonstrates its generalizability. (4) We carried out ablation studies to show the effectiveness of our consistency training approach.

## 2 RELATED WORK

Ronneberger et al. introduced U‐Net [3] for medical image segmentation, which uses an encoder‐decoder structure with symmetrical contracting (down‐sampling) and expansive (up‐sampling) paths, and skip connections, to learn both low and high‐level features. Due to its success, the U‐Net architecture has been widely adopted in medical image segmentation, and similar models [12, 13, 14] that build on this architecture have been proposed.

Jha et al. proposed a model titled ResUNet++ [15] for polyp segmentation in colonoscopy, which is a combination of ResNet [16] and U‐Net [3] architectures. They used residual blocks to prevent the gradient vanishing problem. They also proposed to use Atrous Spatial Pyramid Pooling (ASPP) [17] to capture contextual information within the network and squeeze and excitation blocks [18] to reduce the redundant information.

In another work, Jha et al. [19] connected two U‐Nets, namely, the double U‐Net. The main motivation of double U‐Net is to capture more semantic details [19]. They carried out experiments on different medical image segmentation tasks including colonoscopy, dermoscopy, and nuclei images. They demonstrated that they were able to capture more details by using two connected U‐Nets back to back. Zhou et al. [20] proposed a nested U‐Net architecture. The main idea behind this work is to redesign skip connections to reduce the gap between encoder and decoder layers. The authors tested their model on different medical image segmentation tasks that focus on the segmentation of nuclei, polyps in colonoscopy, lung nodules, and liver, outperforming U‐Net on these datasets.

Huang et al. [6] proposed UNET 3+, a U‐Net based architecture with full‐scale connections. The motivation for this study is to capture details and semantics by combining low and high‐level features at different scales. They used VGG‐16 [21] and ResNet‐101 [16] as backbone. While UNET 3+ showed that full‐scale connections improve segmentation, the following work showed that lower layers are mostly redundant as the higher levels capture both low and high‐level features. Wu et al. [5] proposed using cascaded partial decoder for the problem of salient object detection. Their experiments showed that the third encoder layer carried low‐level features as well as high‐level ones, therefore concatenations of lower layers are mostly redundant. Based on these findings, they developed the partial decoder which does not use the features of the first two encoder layers in the attention module. Using partial decoder and attention module, they outperformed state‐of‐the‐art models. Wei et al. [4] proposed a novel polyp segmentation network titled Shallow Attention Network. Following the findings of Wu et al. [5], the authors ignored the connections from the first two encoder layers. To prevent bias in training, they also proposed a color exchange operation to decouple the image contents and colors. Moreover, they developed a probability correction strategy to increase segmentation accuracy at inference time. Meanwhile, Fan et al. [22] introduced a parallel reverse attention network for polyp segmentation. They reduced the number of parameters required by using a parallel partial decoder. They also proposed to use reverse attention [23] to better capture structural details.

Ding et al. [24] developed asymmetric convolutions which strengthen the square convolution kernels. Asymmetric and basic convolutions were tested separately as part of AlexNet [25] and ResNet [16] architectures, and the asymmetric convolutions were shown to be more successful in image classification tasks. Hu et al. [18] proposed squeeze and excitation networks. The main idea of this network is to weigh each feature map in order to improve the representational power of relevant features. Similarly, Jha et al. [26] proposed a real‐time polyp segmentation model which consists of residual and squeeze and excitation blocks with fewer parameters. Their model demonstrated a significant frame per second (fps) improvement over the state‐of‐the‐art models. Zhao et al. [27] proposed a polyp segmentation model titled MSNET. They developed a Multi‐scale Subtraction Module to reduce inaccurate localization and the problem of blurred edges in polyp segmentation.

Consistency training has been used in semi‐supervised learning to leverage unlabeled data by creating variations of the available data and combining the loss with the loss that comes from training the data available. Ouali et al. [28] proposed cross‐consistency training which improves the encoder's representations through different perturbations for semi‐supervised semantic segmentation. Sohn et al. [29] presented consistency training for image classification. They used pseudo labels with weak and strong augmentations of images. More recently, Wu et al. [30] proposed mutual consistency learning for semi‐supervised medical image segmentation. They used a shared encoder and decoders using different up‐sampling strategies.

In this work, we propose PlutoNet and a novel *consistency training approach*, which ensures a balance between the low‐level salient details at different scales learned through the *modified partial decoder* and the more relevant higher‐level semantic features learned through the auxiliary decoder. Enforcing consistency through a combined loss helps strengthen learned representations, and improves our model's generalizability on unseen datasets and different domains.

## 3 METHOD

An overview of our model is demonstrated in Figure 1. We primarily adopt a lightweight encoder‐decoder structure using the last three encoder layers of EfficientNetB0, followed by the *modified partial decoder*. We apply 64 convolution filters to the output of the encoder layers before they go into the full‐scale connections, which further reduces the number of parameters. In order to handle variations in appearance, we use asymmetric convolutions. Each decoder layer consists of an asymmetric convolution block followed by a squeeze and excitation block. Then we enforce consistency by combining the loss of the *modified partial decoder* and the auxiliary decoder.

### 3.1 Modified partial decoder

Based on the findings of the experiments by Wu et al. [5], Erol et al. [7] removed the full‐scale skip connections of the earlier layers. This way, they combined partial decoder and full‐scale skip connections, namely the *modified partial decoder* at different scales, while reducing the redundant and less informative features of the earlier layers.

(1) acb←relu(bn(conv(3x1))+bn(conv(1x3))+bn(conv(3x3))),

(2) $$d1\leftarrow se(acb\left(c\left(conv\left(e^{3}\right),conv\left(e^{4}\right),conv\left(e^{5}\right)\right)\right)),$$

(3) $$d2\leftarrow se(acb\left(c\left(d^{1},conv\left(e^{3}\right),conv\left(e^{5}\right)\right)\right)),$$

(4) $$d3\leftarrow se(acb\left(c\left(d^{1},d^{2},conv\left(e^{5}\right)\right)\right)).$$

In Equations (2), (3) and (4), c, $d^{1}$, $d^{2}$, $d^{3}$, $e^{3}$, $e^{4}$, $e^{5}$, se, acb, conv represent concatenate, decoder1, decoder2, decoder3, encoder3, encoder4, encoder5, squeeze and excitation, asymmetric convolution block, and convolution, respectively. As mentioned earlier, we skip the connections to the earlier layers $e^{1}$ and $e^{2}$ as the higher layers carry the low‐level features that are already learned through the earlier layers which makes the connections to the two early layers redundant. $e^{3}$ and $e^{4}$ are concatenated with the feature maps learned at the same scale, as well as with feature maps from larger scales. $e^{5}$ is concatenated with all of the three decoder layers. We also concatenate inter‐decoder layers at smaller and larger scales. These connections are demonstrated in Figure 1d. Ding et al. [24] proposed asymmetric convolutions to strengthen kernels, making them able to handle variations in appearance and size. In our work, we use asymmetric convolutions to handle variations in appearance, aspect ratio, and size of the polyps as suggested by Erol et al. [7]. After we enrich the feature space using asymmetric convolutions, we weigh each feature map using a squeeze and excitation block to increase the representation of the more relevant features. This channel‐wise feature recalibration is done at every layer. A detailed view of the Asymmetric Convolution block and the Squeeze and Excitation Block can be seen in Figure 1c. Equation 1 shows the asymmetric convolution block structure. Here, bn represent batch normalization.

### 3.2 Decoder consistency training

Our ablation studies with *modified partial decoder* shows that although each added component increases the performance in segmentation metrics such as Dice and IoU, it decreases precision. This decrease in precision introduces uncertainty in the classification of certain regions as polyps or non‐polyps. In order to overcome this challenge, we propose a novel *consistency training approach* that consists of a shared encoder, the *modified partial decoder*, and the auxiliary decoder that are trained with a combined loss to enforce consistency (Figure 1b). While conventionally used in unsupervised segmentation [28], we use our consistency training approach to ensure a balance between the salient details at different scales learned through the *modified partial decoder* and the more relevant higher‐level semantic features learned through the auxiliary decoder. For the auxiliary decoder, we use the decoder part of the Shallow Attention proposed by Wei et al. [4] which requires few parameters. The auxiliary decoder adds only 200 parameters to our network architecture and is only needed for training. While the *modified partial decoder* learns salient details at different scales, from an enriched feature space extracted using asymmetric convolutions, the auxiliary decoder focuses on more relevant higher‐level semantic features learned through an attention mechanism built on a series of element‐wise multiplications of features extracted only from higher layers. Equation (5) shows how the auxiliary decoder works.

(5) $$d\leftarrow conv(c\left(e^{3}*e^{4}*e^{5},e^{4}*e^{5},e^{5}\right)).$$

ALGORITHM 1 **Consistency training algorithm.**

**Table:** 

$P_{m}\leftarrow f_{m}\left(x,\theta \right)$	▹ $f_{m}$ and $P_{m}$ represent main decoder and its prediction.
$P_{a}\leftarrow f_{a}\left(x,\theta \right)$	▹ $f_{a}$ and $P_{a}$ represent auxiliary decoder and its output.
$L_{c}=1-\left(2*\frac{\sum P_{m}*P_{a}}{\sum P_{m}^{2}+\sum P_{a}^{2}+\varepsilon }\right)$	▹ Consistency Loss
$L_{s}=1-\left(2*\frac{\sum P_{m}*P_{t}}{\sum P_{m}^{2}+\sum P_{t}^{2}+\varepsilon }\right)$	▹ Supervised Loss
$L=L_{s}+\alpha L_{c}$	▹ Total Loss

We enforce consistency by combining the loss of the *modified partial decoder* and the auxiliary decoder, which encourages the outputs of the decoders to be consistent. The algorithm we follow is shown in Algorithm 1. It also shows how we calculate the total loss, where $P_{t}$, $P_{m}$ and $P_{a}$ represents ground truth, the output of the *modified partial decoder* and the output of the auxiliary decoder, respectively.

## 4 EXPERIMENTAL DETAILS

We evaluated our model extensively for the segmentation of polyps in colonoscopy and wireless endoscopy images on five different public datasets and carried out an ablation study to show the effectiveness of our decoder consistency training approach. We followed the experimentation set‐up suggested by Fan et al. [22]; we split Kvasir‐SEG [2] and CVC‐ClinicDB [8] datasets as 80% training, 10% validation, and 10% testing, and carried out ablation studies on the Kvasir‐SEG dataset. Then we tested our model further on the unseen ETIS [9], EndoScene [10], and CVC‐ColonDB [11] datasets. ETIS is a dataset consisting of images captured by capsule endoscopy and differs greatly in resolution. The datasets we used to evaluate our model and the dataset properties are summarized in Table 1. We implemented our model in TensorFlow accelerated by NVIDIA RTX 3050TI 4GB. All images are resized to 224 x 224 x 3. We used random rotation and horizontal flip data augmentation techniques. We set up an early stopping scheme according to the validation loss (trained for 30 epochs). We set the initial learning rate to 1e−4 and used the Adam optimizer.

**TABLE 1:** Datasets we experimented on for polyp segmentation along with their properties.

Dataset	# images	Image size	Application
Kvasir SEG [2]	1000	Variable	Colonoscopy
CVC‐ClinicDB [8]	612	384 x 288	Colonoscopy
CVC‐ColonDB [11]	380	574 x 500	Colonoscopy
EndoScene [10]	60	574 x 500	Colonoscopy
ETIS [9]	196	1225 x 966	Endoscopy

## 5 RESULTS

We compared PlutoNet's performance to a benchmark consisting of the state‐of‐the‐art models, namely, UNet [3], UNet++ [20], SFA [31], PraNet [22], MSNet [27] and Shallow Attention (SANet) [4]. Table 2 shows our model's results compared to the results of the benchmark studies. In order to evaluate the computation and memory requirements of our model in comparison to the benchmark, we also provide FLOP (floating‐point operation) and number of parameters metrics. FLOP is a metric used to measure the amount of computation a model needs to perform for its forward pass, and it provides an objective measure of the computational complexity of a deep learning model, independent of hardware or implementation specifics. Our experiments demonstrate that our model has a lower FLOP requirement, and it needs less than 10% of the parameters required by its counterparts.

**TABLE 2:** A comparison of our model's performance to the state‐of‐the‐art polyp segmentation models is demonstrated. FLOP and number of parameters are also added to evaluate the computation and memory requirements of our model in comparison to the benchmark. Our model performs particularly well on unseen datasets and datasets across different domains, while requiring less computation and memory compared to its counterparts.

	Kvasir		ClinicDB		ColonDB		EndoScene		ETIS		Param	FLOP
Methods	Dice	IoU	Dice	IoU	Dice	IoU	Dice	IoU	Dice	IoU	Million	GMac
UNet	0.818	0.746	0.823	0.755	0.512	0.444	0.710	0.627	0.398	0.335	15.7M	—
UNet++	0.821	0.743	0.794	0.729	0.483	0.410	0.707	0.624	0.401	0.344	9M	—
SFA	0.723	0.611	0.700	0.607	0.469	0.347	0.467	0.329	0.297	0.217	—	—
PraNet	0.898	0.840	0.899	0.849	0.709	0.640	0.871	0.797	0.628	0.567	30.5M	13.11
MSNET	**0.907**	**0.862**	**0.921**	**0.879**	**0.755**	**0.678**	0.869	0.807	0.719	0.664	25.2M	16.97
SANet	0.904	0.847	0.916	0.859	0.753	0.670	0.888	0.815	0.750	0.654	23.9M	11
Ours	0.895	0.811	0.909	0.832	0.694	0.531	**0.919**	**0.851**	**0.829**	**0.709**	**2.6M**	**9**

Our model outperformed UNet, UNet++, and SFA on all datasets for Dice and IoU metrics. Even though we only used about less than 10% of the parameters required by PraNet, MSNet, and Shallow Attention, our model outperformed state‐of‐the‐art models on the unseen datasets of ETIS with an 82.9% Dice score and on EndoScene with a 91.9% Dice score. It should be noted that PlutoNet performs significantly better than the state‐of‐the‐art models on unseen datasets. Among these unseen datasets; ETIS [9], EndoScene [10], and CVC‐ColonDB [11], PlutoNet outperforms all benchmark models on two of them for both Dice and IoU metrics. Particularly, PlutoNet outperforms all other models with a large margin on ETIS, which is a dataset of images captured by capsule endoscopy and differs greatly in resolution. This supports PlutoNet's ability to learn stronger representations that generalize well to unseen datasets and datasets across different domains.

### 5.1 Ablation study

**FIGURE 2:**
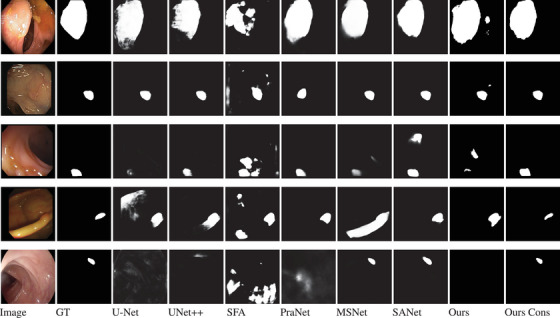
Sample segmentation results of the benchmark models compared with PlutoNet with and without our *consistency training approach*. Images shown in the first column, from top to bottom, belong to Kvasir, ClinicDB, ColonDB, EndoScene, and ETIS datasets, respectively. (Ours stands for PlutoNet without consistency, Ours Cons stands for PlutoNet with our *consistency training approach*.).

To show the effectiveness of our *consistency training approach*, we compared our model with and without our consistency training approach. Table 3 shows the results of our model's performance with and without consistency training. Using our consistency training approach, we are able to reduce false positive rates and improve the segmentation results for Kvasir‐SEG [2], ClinicDB [8], ETIS [9], and EndoScene [10] datasets. Sample segmentation results as seen in Figure 2 also support these improvements.

**TABLE 3:** Ablation study showing the effectiveness of consistency training. Our consistency training approach reduces false positive rates and improve the segmentation results in general (Cons stands for Consistency).

Metric	Kvasir	Kvasir Cons	ClinicDB	ClinicDB Cons	ColonDB	ColonDB Cons	ETIS	ETIS Cons	EndoScene	EndoScene Cons
Dice	0.8839	**0.8954**	0.8964	**0.9085**	**0.7178**	0.6935	0.8042	**0.8296**	0.8979	**0.9192**
IoU	0.7920	**0.8105**	0.8122	**0.8323**	**0.5598**	0.5308	0.6725	**0.7088**	0.8147	**0.8506**
Precision	0.9250	**0.9559**	0.9492	**0.9515**	0.7923	**0.8845**	0.7929	**0.8343**	0.8905	**0.9221**
Recall	**0.8315**	0.8265	0.8451	**0.8662**	**0.6556**	0.5702	0.8165	**0.8255**	0.9024	**0.9186**

**FIGURE 3:**
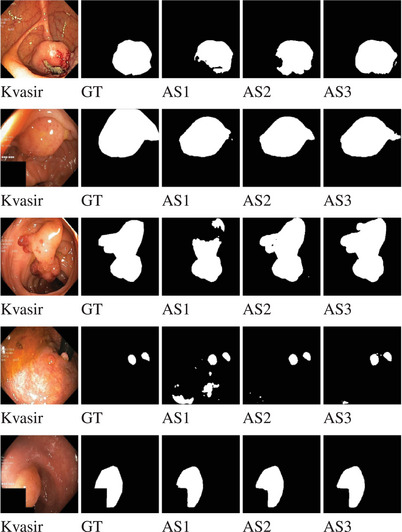
As part of our ablation studies, sample outputs of each component on the Kvasir dataset [2] are shown. AS1, AS2, and AS3 represent Ablation Study 1, Ablation Study 2, and Ablation Study 3, respectively. AS1 contains Backbone and *modified partial decoder*. Please note how using an asymmetric convolution block (AS2) instead of a conventional convolution block (AS1) improved the segmentation output by reducing the number of false positives, in other words, the pixels that were segmented as polyps by mistake. An example of this can be clearly observed in the fourth row. Adding squeeze and excitation block (AS3) captured more semantic details reducing false negatives and leading to more accurate segmentation.

We carried out another ablation study on Kvasir dataset to evaluate the effectiveness of each added component of PlutoNet architecture. First, we used EfficientNetB0 as the backbone with *modified partial decoder*. We achieved an 87.88% Dice and a 78.38% IoU score. Then, in addition to the first component, we added the asymmetric convolution block instead of using the basic convolution block. We reported an improvement of 0.51% Dice and 0.82% IoU score. Figure 3 demonstrates that by adding asymmetric convolutions we were able to capture more semantic information by improving true positive rates. Then, we integrated the squeeze and excitation block into the previous model. With a combination of asymmetric convolution and squeeze and excitation block, we achieved a 90.97% Dice and an 83.45% IoU score. This led to an improvement of a 2.58% Dice and a 4.25% IoU score. Results of our ablation study can be found in Table 4, while sample outputs of each component of the Kvasir dataset are shown in Figure 3.

**TABLE 4:** Ablation study on the Kvasir dataset [2] to evaluate the effectiveness of each component of our model. Backbone represents EfficientNetB0 in our experiments. PD, FSC, ACB, and SE represent Partial Decoder, Full‐Scale Connection, Asymmetric Convolution Block, and Squeeze and Excitation, respectively. AS1, AS2, and AS3 stand for Ablation Study 1, Ablation Study 2, and Ablation Study 3, respectively. Using an asymmetric convolution block (AS2) instead of the conventional convolution block (AS1) improved Dice, IoU, AUC, and recall scores. Using Squeeze and Excitation block with the asymmetric convolution block (AS3) achieved the best Dice, IoU, AUC, and recall scores, which underlines the effectiveness of these components.

Ablation study	Dice	IoU	AUC	Precision	Recall	Parameter Sizes
Backbone + PD + FSC (AS1)	0.8788	0.7838	0.8951	**0.9534**	0.7979	2,192,545
Backbone + PD + FSC + ACB (AS2)	0.8839	0.7920	0.9046	0.9440	0.8188	2,620,961
Backbone + PD + FSC + ACB + SE (AS3)	**0.9097**	**0.8345**	**0.9306**	0.9380	**0.8727**	2,626,337

### 5.2 Limitations and future work

Although our model achieves state‐of‐the‐art results, there are some limitations to it. By ignoring lower‐level features, we are able to largely decrease redundant information, however, we might be missing tiny polyps. This is a trade‐off in order to reduce the number of parameters and false positives. Enforcing consistency by combining the loss of the *modified partial decoder* and the auxiliary decoder, we are able to improve the encoder's representations without losing learned relevant semantic details in most cases. In Table 3, we see that the Precision is higher as the false positive rate is much lower for all experiments that span five different datasets; however, the Recall is noticeably lower for the ColonDB dataset which suggests an increase of false negatives. In all other experiments with the remaining four datasets, we see that the Recall is higher or comparable to the state‐of‐the‐art with consistency training.

## 6 CONCLUSION

Colon cancer is preventable with early intervention. Recent advances in deep learning models are used to minimize the number of polyps that go unnoticed during colonoscopy, and to accurately segment the detected polyps. However, these models often require high computation and memory, which may pose a problem with real‐time applications. We propose a novel polyp segmentation model titled PlutoNet, to address these challenges. PlutoNet requires only 9 FLOPs and 2,626,537 parameters in test time while outperforming state‐of‐the‐art models on several datasets. We perform ablation studies and experiments which show that PlutoNet performs significantly better than the state‐of‐the‐art models, particularly on unseen datasets and on datasets across different domains. Our experiments span five different datasets for polyp segmentation in colonoscopy and wireless capsule endoscopy images. Our model outperformed UNet, UNet++, and SFA on all datasets for Dice and IoU metrics. Even though we used about less than 10% of the parameters required by PraNet, MSNet, and Shallow Attention, our model outperformed state‐of‐the‐art models on the ETIS dataset with an 82.9% Dice score and on EndoScene dataset with a 91.9% Dice score. It should be noted that both ETIS and EndoScene are unseen datasets, that is, they are not used for training but only for testing. Moreover, ETIS consists of images captured by capsule endoscopy and differs greatly in resolution. The performance of our model on these datasets underline the generalizability of our model, thanks to the strengthened representations learned through our novel *consistency training approach*.

## AUTHOR CONTRIBUTIONS

**Tugberk Erol**: Conceptualization; methodology; software; validation; visualization; writing—original draft; writing—review and editing. **Duygu Sarikaya**: Conceptualization; methodology; software; supervision; validation; visualization; writing—original draft; writing—review and editing.

## CONFLICT OF INTEREST STATEMENT

The authors declare no conflicts of interest.

## Data Availability

All datasets used are publicly available and can be accessed online.
